# The Management of Pregnancy in Women with Hepatocellular Adenoma: A Plea for an Individualized Approach

**DOI:** 10.1155/2012/725735

**Published:** 2012-12-24

**Authors:** Mirelle E. E. Bröker, Jan N. M. Ijzermans, Susanna M. van Aalten, Robert A. de Man, Türkan Terkivatan

**Affiliations:** ^1^Department of Surgery, Erasmus Medical Center, P.O. Box 2040, 3000 CA Rotterdam, The Netherlands; ^2^Department of Gastroenterology and Hepatology, Erasmus Medical Center, P.O. Box 2040, 3000 CA Rotterdam, The Netherlands

## Abstract

Because of the risk of hormone-induced growth and spontaneous rupture of hepatocellular adenoma (HCA) during pregnancy, special considerations are required. Due to the scarcity of cases, there is no evidence-based algorithm for the evaluation and management of HCA during pregnancy. We think it should be questioned if it is justified to discourage pregnancy in all women with HCA. The biological behavior of this benign lesion might be less threatening than presumed and a negative advice concerning pregnancy has great impact on the lives of these young female patients. The balance between the pros and cons of hepatic adenomas and pregnancy should be reconsidered. In our center, pregnancy in women with an HCA up to 5 cm is no longer discouraged in close consultation with the patient, her partner, and members of the liver expert team.

A strong association between hepatocellular adenoma (HCA) and the use of oral contraceptives (OC) was first described in 1973 [1]. The hypothesis that there is a relation between steroids and HCA has been supported by many authors but is still not understood [2–4]. Due to the increased levels of endogenous hormone production, which may cause hormone-induced growth and rupture, HCA requires special attention during pregnancy [5, 6]. Patients with a growing or ruptured HCA mostly present themselves with persistent or acute severe pain localized in the upper right quadrant and in the epigastric region. In the literature, the maternal and fetal mortality risks of ruptured HCA during pregnancy has been reported to be 44 and 38% respectively [7]. However, all these cases were published in the 1970s or 1980s, in which there might have been a delay in diagnosis as the entity of ruptured HCA was not well known and less advanced imaging methods were used. 

In the recent years the widespread use of highly advanced image modalities has probably decreased the delay in the diagnosis of HCA and the associated maternal and fetal mortality significantly. Because of the unpredictable behavior of HCA during the increased levels of endogenous hormones, we used to advise women with a large HCA or a growing and hormone-sensitive HCA to avoid pregnancy, as most other experts in this field do [6, 8]. Even if HCA was incidental findings previous to a pregnancy without having caused any complications, women were still advised not to get pregnant as long as the HCA is present. Because of the overall agreed advice to avoid pregnancy in patients with HCA, the diagnosis of HCA has severe impact on the lives of these young fertile women. 

As to date, there are limited data about the behavior of HCA during pregnancy and labor. 

From the international literature between 1966 and 2003, Cobey and Salem retrieved 26 cases of women presenting with HCA during pregnancy or early postpartum and proposed an algorithm for their diagnosis and management [7]. Presentation was acute and often dramatic with rupture of the adenoma in 16 women and frequently with a delay in establishing the correct diagnosis, with high maternal and fetal mortality (44% and 38%, resp.). The hormone-induced growth and risk of rupture seemed to be the highest during the third trimester of pregnancy, most probably because of the cumulating level of estrogens and an increase in the hyperdynamic circulation combined with an increase in vascularity of the liver [7]. An aggressive approach towards resection of HCA was advocated, especially for those greater than 5 cm. Small adenomas were supposed to be managed by observation [7]. It is important to realize that most of these reports were published in a time period during which this disease entity was relatively unknown and treatment in an emergency setting was less advanced. 

In our hospital, we monitored 12 women with one or more documented HCAs during a total of 17 pregnancies. In four cases, HCA grew during pregnancy, requiring a Caesarean section in one patient (two pregnancies) and RFA in one patient during the first trimester of pregnancy because of significant growth of the adenoma. All pregnancies had an uneventful course with a successful maternal and fetal outcome [9]. We concluded not to discourage all women with HCA from pregnancy. In our tertiary referral center, we closely observe pregnant women with a HCA smaller than 5 cm in a clinical trial [10]. In this study, the size of the lesion is an exclusion criterion when exceeding 5 cm, but the number of HCAs present in the liver is not. Three studies investigated the association between the risk of rupture and the number of HCAs [11]. This risk did not differ between single and multiple HCAs [12–14]. In our previous study, the number of HCAs in the women observed during pregnancy varied between 1 and more than 10 HCAs. We concluded that only in women with large tumors and a complicated pregnancy previously, pregnancy should be discouraged [9]. 

Furthermore, in our opinion, none of the subgroups from the molecular and pathological subtype classification of the Bordeaux group legitimizes objection against pregnancy. Although the number of cases described in literature is small, no difference has been demonstrated in the risk of bleeding between the two major subgroups, the inflammatory and the hepatocyte nuclear factor 1*α*-inactivated HCAs [15, 16]. 

If women have large tumors or have experienced complications of HCA in previous pregnancies, an intervention (surgery, RFA, embolisation) should be recommended before pregnancy. Moreover, in 2006 we reported a series of 48 patients in which 44% of HCA were discovered after the patient had sustained at least one pregnancy [17].

Intervention during pregnancy may be associated with greater risk for both mother and child. The Society of American Gastrointestinal and Endoscopic Surgeons (SAGES) provided guidelines for diagnosis, treatment, and use of laparoscopy for surgical problems during pregnancy [18, 19]. In one in 635 pregnancies, a nonobstetric operation, in particular appendectomy, cholecystectomy, and adnexal procedures, is required during pregnancy [20]. These guidelines suggest that the laparoscopic approach should be preferred instead of laparotomy in most abdominal operations. 

The maternal and fetal outcomes following abdominal surgery in pregnancy improved over last decade but the exact risk of HCA-related interventions during pregnancy to both mother and fetus is unknown [21]. Abdominal surgery may be more difficult during pregnancy in the late second and third trimester because of the limited wideness in the upper abdomen due to the enlarged uterus and risk of steatotic changes of the liver in these patients. General anesthesia seems to have the least risk in the 2nd trimester of pregnancy [5]. The role of RFA during pregnancy is not yet been studied extensively. In our previous study, we described a RFA procedure during the first trimester of pregnancy [9] and a pregnant patient with a HCA which was treated by RFA during her second trimester of pregnancy (18th week of gestation) was reported by Fujita et al. [22]. After systematically reviewing the literature, Wilson et al. suggested that angioembolization and formal resection in case of hemorrhage of HCA during pregnancy is safe for both the mother and the fetus with good clinical outcomes [23]. We believe that selective arterial embolization should only be used as a live-saving treatment in those cases where RFA or surgery is inadequate or too risky to control the bleeding adenoma. The increased risk of radiation exposure to the fetus, especially before 26 weeks of gestation [24, 25], should be avoided if possible. 

Because HCA might have the tendency to rupture during delivery, some authors suggest a Caesarean section (C-section). In our study three C-sections (two patients) were performed, without complications. In one case the C-section was performed in consultation with the patient because of marked growth and an unknown risk of rupture of the HCAs. In the other, C-section was due to decelerations on the cardiotocography [9]. All other patients had a normal delivery without complications. Therefore, in our opinion patients with HCA may deliver vaginally if there are no complicating factors, like perinatal problems.

In conclusion, it seems to be justified that a pregnancy should be discouraged in patients with a large HCA (>5 cm) or those who experienced complications of the lesion in previous pregnancies (Figure 1). In those cases a surgical resection, RFA, or embolisation should be recommended before pregnancy. In our center we do not discourage pregnancy in women with a HCA <5 cm (Figure 2) if they accept the risk of interventions in case of growth of the adenoma. Close guidance of these women and monitoring of the hepatic adenoma by liver ultrasound every 6 weeks during pregnancy are strongly advocated [10, 26]. 

## Figures and Tables

**Figure 1:**
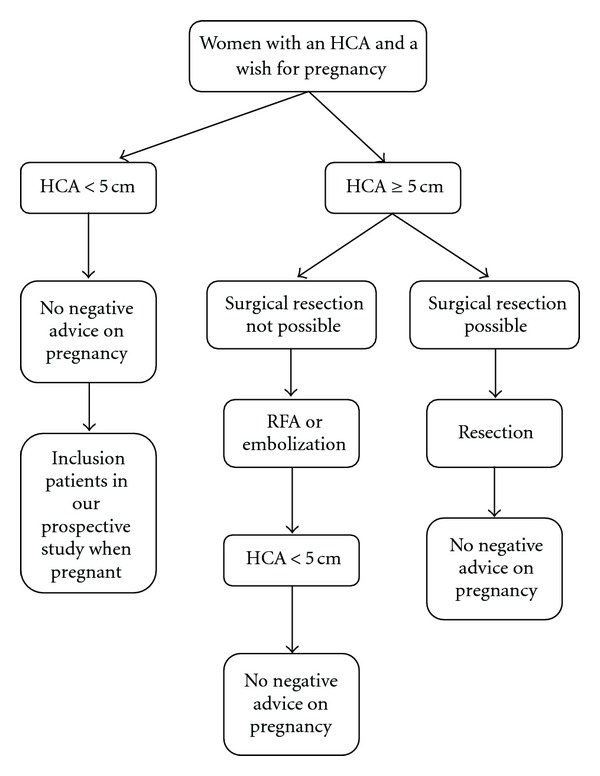
Flowchart for women with a HCA and a wish for pregnancy.

**Figure 2:**
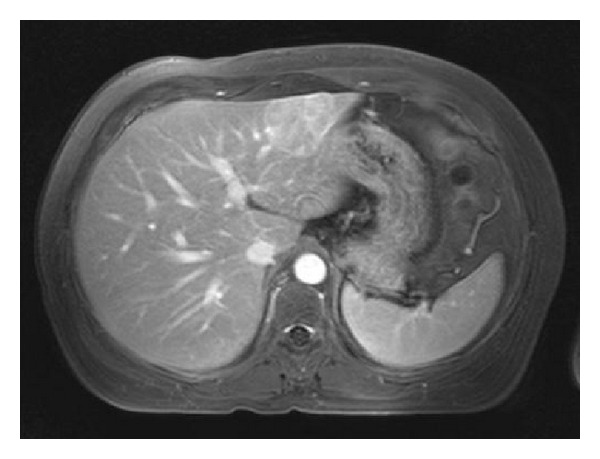
An example of a woman with a HCA of 4.2 cm in segment 2/3 in which pregnancy will not be discouraged.
